# NT-proBNP as a predictor of death and cardiovascular events in patients with type 2 diabetes

**DOI:** 10.1186/s13098-022-00837-6

**Published:** 2022-05-03

**Authors:** Marcus Vinicius Bolivar Malachias, Magnus Olof Wijkman, Marcello Casaccia Bertoluci

**Affiliations:** 1 Department of Internal Medicine, Faculdade Ciências Médicas de Minas Gerais, Fundação Educacional Lucas Machado, Alameda Ezequiel Dias, 275, Centro, Belo Horizonte, MG 30130-110 Brazil; 2 Department of Internal Medicine and Department of Health, Medicine and Caring Sciences, Linköping University, Norrköping, Sweden; 3 Internal Medicine Department, Faculdade de Medicina, Universidade Federal do Rio Grande do Sul, Porto Alegre, RS Brazil; 4 Endocrinology Unit, Hospital de Clínicas de Porto Alegre, Porto Alegre, RS Brazil

**Keywords:** Natriuretic peptides, Biomarkers, Diabetes, type 2, Risk assessment, Cardiovascular disease, Prognosis, Morbidity

## Abstract

Existing risk prediction scores based on clinical and laboratory variables have been considered inaccurate in patients with Type 2 Diabetes Mellitus (T2DM). Circulating concentrations of natriuretic peptides have been used to aid in the diagnosis and to predict outcomes in heart failure. However, there is a growing body of evidence for the use of natriuretic peptides measurements, mainly N-terminal pro-B-type natriuretic peptide (NT-proBNP), as a tool in risk stratification for individuals with T2DM. Studies have demonstrated the ability of NT-proBNP to improve outcomes prediction when incorporated into multivariate models. More recently, evidence has emerged of the discriminatory power of NT-proBNP, demonstrating, as a single variable, a similar and even superior ability to multivariate risk models for the prediction of death and cardiovascular events in individuals with T2DM. Natriuretic peptides are synthesized and released from the myocardium as a counter-regulatory response to increased cardiac wall stress, sympathetic tone, and vasoconstriction, acting on various systems and affecting different biological processes. In this article, we present a review of the accumulated knowledge about these biomarkers, underscoring the strength of the evidence of their predictive ability for fatal and non-fatal outcomes. It is likely that, by influencing the functioning of many organs, these biomarkers integrate information from different systems. Although not yet recommended by guidelines, measurement of natriuretic peptides, and particularly NT-proBNP, should be strongly considered in the risk stratification of individuals with T2DM.

## Background

Individuals with diabetes mellitus (DM) are at a higher risk for cardiovascular disease (CVD) and death from any cause when compared to people without DM [1]. Different risk stratification models have been proposed for individuals with DM given their high heterogeneity [2]. Validated models such as the Framingham risk score (FRS), the United Kingdom Prospective Diabetes Study (UKPDS), The Systematic Coronary Risk Evaluation (SCORE) project risk scores for fatal Coronary Heart Disease (CHD) and CVD, Diabetes Epidemiology Collaborative Analysis of Diagnostic Criteria in Europe (DECODE) showed limited ability to stratify the risk of mortality and cardiovascular events in individuals with DM [3, 4].

Cardiology and diabetes societies have suggested different approaches to address cardiovascular risk in individuals with Type 2 DM (T2DM). The European Society of Cardiology (ESC) and the European Association for the Study of Diabetes (EASD) have suggested that treatment decisions should be based on a 10-year risk stratification model for fatal CVD adapted from the European Guidelines on CVD prevention in clinical practice [5]. The American Diabetes Association (ADA) 2022 guidelines recommend the American College of Cardiology (ACC)/American Heart Association (AHA) atherosclerotic cardiovascular disease (ASCVD) risk calculator (Risk Estimator Plus) as a useful tool to estimate the risk of ASCVD at 10 years [6]. These approaches have limitations by not distinguishing important variables such as duration of disease, diabetes complications, and degree of glycemic control [6]. These guidelines, however, recognize the potential benefits of incorporating new biomarkers in the risk stratification of patients with DM [6].

Natriuretic peptides (NP) are biomarkers of myocardial stress that have been used to aid in the diagnosis, assessment of disease severity, and prognosis in heart failure (HF) [7, 8]. There is a growing body of evidence for using NP levels as a sensitive risk stratification tool also for individuals with T2DM. It is promising to use these biomarkers either as a single variable [9–12] or added to multivariate models [13, 14] in the prediction of CV events and death in individuals with T2DM. This review aims to discuss the physiological and pathophysiological mechanisms of NP and the potential clinical benefit of incorporating these biomarkers to improve the stratification of risk of death and CV outcomes in individuals with T2DM.

## Physiology and pathophysiology of natriuretic peptides

There are three main biologically active NP: atrial NP (ANP), brain NP (currently called B-type NP, BNP) and C-type NP (CNP) [15]. Under normal conditions, the main sites of synthesis and secretion of ANP and BNP are the cardiac atria. However, in pathological states, the ventricles start to produce and release higher concentrations of BNP. Although ANP are 50-fold greater, under physiological conditions, BNP has intensely more pronounced activity and, under pathological conditions, its plasma concentrations become 5–10 times greater than that of ANP [15]. ANP and BNP preferentially bind to the NP receptor A (NPRA). All three NP—ANP, BNP, and CNP—can bind to the NP receptor C (NPRC), which, because it lacks a catalytic domain of guanylyl cyclase, works as an NP scavenging receptor [15]. Binding of ANP and BNP to NPRA produces intracellular second messenger cyclic guanosine monophosphate which activates effector molecules such as cGMP-dependent protein kinase, gated ion channels, and phosphodiesterases to induce physiological actions in target tissues, including salt and water excretion, vasodilation, anti-inflammation, anti-hypertrophy, anti-fibrosis, anti-apoptosis, immunosuppressive responses and inhibition of the renin–angiotensin–aldosterone system (RAAS) [16]. CNP is an autocrine and paracrine mediator released by endothelial cells and binds to the NP receptor B (NPRB) [15]. CNP acts predominantly on the endothelium playing a significant role in vascular homeostasis and may influence coronary circulation, and blood pressure, in addition to actions on the central nervous system and bone growth [17].

BNP has a longer half-life (12 to 20 min) than ANP (0.5 to 4 min) and exhibits more pronounced natriuretic and diuretic effects [15]. CNP, in turn, has very low plasma concentrations and a short half-life (2 to 3 min) [15]. NT-proBNP, an inactive terminal molecule, despite being released in equimolar proportions to BNP, has greater plasma stability and a longer half-life (90 to 120 min), being considered the preferred laboratory marker [15].

Urodilatin is an intrarenal NP, synthesized in the renal cortical tubules around the collecting ducts, identified in the urine, but not in the blood, with paracrine action, via renal NPRA, in the regulation of sodium and water volume and inhibition of the RAAS [15]. Many other NP were found in venoms of different snake species, such as Dendroaspis NP (DNP) isolated from *Dendroaspis angusticeps* (green mamba), but not yet clearly evidenced in humans [18].

NP act in addition to the heart, arteries, and veins, in several regulatory mechanisms involving the kidneys, central nervous system, pancreas, liver, adipose tissue, and skeletal muscle, being, therefore, able to reflect a wide range of changes in physiological homeostasis [15, 19].

## NP in the regulation of energy metabolism and diabetes

The energy used in cardiac contractility is predominantly derived from the oxidation of fatty acids in mitochondria, which are abundant in the myocardium. But the heart also uses additional energy sources, such as carbohydrates, lipids, amino acids, and ketone bodies [19]. In pathological cardiac hypertrophy, there is a reduction in fatty acid oxidation, and an increase in glucose uptake and glycolysis. In T2DM patients, insulin resistance limits the entry of glucose into cardiomyocytes for energy generation, and there is also an increase in fatty acid oxidation, lipid accumulation, and increased oxygen consumption, which compromise cardiac function [19].

NP act in the regulation of energy balance by activating mitochondrial oxidative metabolism, lipid oxidation in skeletal muscle, thermogenic function of brown adipose tissue, further promoting the browning of white adipocytes, increased energy expenditure, and inhibition of food intake [19].

Under physiological conditions, NP increase insulin secretion and sensitivity. In humans, low plasma BNP concentrations have been associated with increased metabolic risk factors and T2DM while higher concentrations of NP, within the physiological range, were associated with a favorable body fat profile, with less visceral fat, reduced insulin resistance, and a lower risk of developing T2DM [19]. In situations of cardiac stress, as well as in several systemic pathological conditions, there is excessive production of ANP and BNP, as a compensatory mechanism [15]. The measurement of high concentrations of NP warns of CV instability, especially in individuals with T2DM [20–22].

## Effect of clinical comorbidities on NP levels

Many clinical variables can affect the circulating levels of NP. The evaluation of NP concentrations should consider age, sex, clinical comorbidities, and pharmacotherapy.

### Age, sex, and race

Circulating BNP concentrations increase progressively with age and are significantly higher in women [23]. There may also be variations in NP concentrations related to race and ethnicity. African Americans have been shown to have lower levels of NT-proBNP than Caucasians [23, 24].

### Obesity

The causes of the inverse association between NP and body mass index (BMI) are not definitively established. The clearance receptor NPRC and neutral endopeptidases are abundant in adipose tissue and may determine, respectively, increased clearance and degradation of BNP. However, peripheral clearance of NT-proBNP is not based on NPRC activity or neutral endopeptidase degradation. Apparently, in people with high BMI values, there is less synthesis and release of NP in cardiomyocytes [25]. Many mechanisms corroborate such low concentrations of NP in obese individuals, including genetics, African ancestry, increased androgens in menopausal women, hyperinsulinemia, insulin resistance, and hypercortisolism [25, 26]. As hyperinsulinemia is often seen in obese individuals, high insulin levels inhibit NP secretion and activity [26]. Low NP concentrations, in turn, can lead to insulin resistance, glucose intolerance, increased metabolically unhealthy adipose tissue, more fluid retention, and increased risk of developing T2DM, hypertension, and CVD [25–27].

## Risk of new-onset diabetes

NT-proBNP is inversely associated with the risk of developing new-onset T2DM. In the EPIC (*European Prospective Investigation into Cancer and Nutrition*)-*Potsdam* study, a population cohort, there was an inverse association between NT-proBNP concentrations and future risk of T2DM [28]. Similar observations were described in the ARIC (*The Atherosclerosis Risk in Communities*) study, in which the risk of incident diabetes was significantly higher in participants with low NT-proBNP levels (< 31 pg/mL) [29]. However, in the *Malmö Diet and Cancer Study*, lower levels of mid-regional ANP, but not NT-proBNP, predicted a higher risk for incident diabetes and larger longitudinal increases in fasting glucose concentrations [30].

## Can measurements of NP guide treatment in patients with diabetes?

There is initial evidence about the usefulness of NP to guide therapy. In the PONTIAC (*NT-proBNP selected prevention of cardiac events in a population of patients with DM without a history of cardiac disease*) study, patients with T2DM without clinical evidence of CVD and with NT-proBNP levels above 125 pg/mL were randomized to receive standard management or intensification of primary prevention with inhibitors of RAAS and beta-blockers, revealing a significant reduction in hospitalization and death due to heart disease after 2 years in the intervention arm [31]. This suggests that patients with T2DM who have elevated NT-proBNP levels appear to benefit from intensified preventive treatment. However, since NT-proBNP elevation was an inclusion criterion, the PONTIAC study did not demonstrate whether the benefit of intensified treatment is larger in patients with T2DM and NT-proBNP elevation than in patients with T2DM who have normal NT-proBNP concentrations.

In a posthoc analysis of the ALTITUDE (*The Aliskiren Trial in Type 2 Diabetes Using Cardiorenal Endpoints*) trial, a significant interaction was observed between baseline NT-proBNP concentrations and the aliskiren treatment, suggesting a harmful effect of renin inhibitor relative to placebo in participants with the highest levels of NT-proBNP due to likely volume overload [32].

An increased risk of hospitalization for HF (HFH) has been observed among patients with T2DM and elevated NT-proBNP levels who received saxagliptin-based treatment [40]. In the SAVOR (*Saxagliptin Assessment of Vascular Outcomes Recorded in Patients with Diabetes Mellitus*) trial, patients in the highest quartile of NT-proBNP (333–46.627 pg/mL) had an increased absolute risk of HFH with saxagliptin use, when compared to those in the lowest quartiles [33].

In an analysis of the CANVAS (*Canagliflozin Cardiovascular Assessment Study*), a baseline NT-proBNP ≥ 125 pg/mL was a predictor of HFH, death from HFH/CV, and all-cause death in high-risk patients with T2DM. Canagliflozin demonstrated greater reductions in the absolute risk of CV outcomes in patients with NT-proBNP levels ≥ 125 pg/mL than in patients with lower concentrations of this biomarker. Mediation analyzes showed that 10.4% of the benefits of canagliflozin in reducing HFH were reflected in the reduction of NT-proBNP [34].

The ongoing PONTIAC II, a prospective randomized trial, will evaluate the effect of high-dose RAAS antagonists and beta-blocker treatment in the primary prevention of cardiac events in T2DM patients with no evidence of preexisting cardiac disease, focusing on the interaction between NT-proBNP concentrations and treatment effects. Based on the PONTIAC I findings, the authors will attempt to demonstrate the superiority of treatment in reducing unplanned hospitalization or death from cardiac events in patients with T2DM and with NT-proBNP > 125 pg/mL [35].

Another ongoing study, the APORT (Asian Diabetes Outcomes Prevention Trial), seeks to identify T2DM patients at high CV risk using elevated NT-proBNP (> 125 pg/mL) to assess intensified therapy with RAAS antagonists, beta-blockers, and sodium–glucose cotransporter 2 (SGLT2) inhibitors in the primary prevention of CV events [36].

## Impact of diabetes treatment on NP concentrations

### Metformin

To identify predictors of increased serum NT-proBNP levels in patients with T2DM, a study that included 185 patients treated with oral antidiabetic drugs and/or insulin showed that, in multivariate analysis, metformin was a negative predictor of increased NT-proBNP [37]. However, a meta-analysis of four studies, that evaluated the effect of metformin on BNP concentrations in patients with T2DM and CHD, concluded that the difference between patients on metformin versus non-metformin therapy was negligible [38].

### Pioglitazone

In a study involving 30 patients with T2DM without evidence of HF, pioglitazone induced significant increases in BNP levels, and basal BNP levels above the upper normal limit were associated with significant additional increases in this biomarker concentration and a reduction in left ventricular ejection fraction [39]. In a subsequent study involving 44 patients, pioglitazone induced volume overload and an increase in NT-proBNP levels without, however, worsening the left ventricular function [40].

### DPP4 inhibitors

In an analysis of 9 trials involving 3056 patients with T2DM, dipeptidyl peptidase-4 inhibitors (DPP-4i) showed no significant effect on modulating BNP or NT-pro-BNP [41].

### SGLT2 inhibitors

SGLT2 inhibitors may reduce NT-proBNP concentrations in patients with T2DM [42, 43], although smaller studies have revealed conflicting results [44, 45]. In the DAPA-HF (*Dapagliflozin And Prevention of Adverse Outcomes in Heart Failure*) trial, participants with T2DM had a significant decrease in NT-proBNP concentrations in the dapagliflozin group, while the placebo group experienced a significant increase [46]. Dapagliflozin significantly reduced BNP in patients with concentrations ≥ 100 pg/mL, but not at lower levels in a study involving a small group of patients with T2DM and stable HF [46]. In EMPEROR-Reduced (*Empagliflozin Outcome Trial in Patients with Chronic Heart Failure with Reduced Ejection Fraction*), higher baseline NT-proBNP concentrations were associated with a higher risk of adverse HF or renal outcomes [47]. Empagliflozin reduced NT-proBNP concentrations by 13% when compared to placebo, but the improvement in the composite endpoint of death and HFH occurred independently of the baseline NT-proBNP concentrations. The concentration of NT-proBNP after treatment with empagliflozin correlated better with subsequent prognosis than did pretreatment levels [47].

### GLP-1 RA

A small study with patients with T2DM and obesity showed that after 12 weeks of treatment with the glucagon-like peptide 1 receptor agonist (GLP-1 RA) liraglutide, there was a significant reduction in body weight, waist circumference, total fat, and lean mass, fat percentage, abdominal visceral adipose tissue areas (VAT), and subcutaneous adipose tissue areas (SAT), and that plasma concentrations of ANP and BNP increased significantly [48]. There were significant correlations between reductions in body composition and increases in plasma levels of ANP and BNP [55]. This is consistent with a previously observed inverse relationship between obesity and NP concentrations [34]. In patients with heart failure with reduced ejection fraction (HFrEF) and T2DM on the other hand, liraglutide decreased ANP and BNP significantly (27% and 25%, respectively), whereas no change was observed in patients without T2DM [49].

### Sulfonylureas

One study found that increasing doses of sulfonylureas were positively associated with increasing concentrations of NT-proBNP. However, patients on combination therapy with sulfonylureas and metformin had lower concentrations of NT-proBNP than those receiving sulfonylureas alone [50].

### Insulin

In a cross-sectional study, women with gestational DM on insulin use had lower NT-proBNP circulating concentrations than those observed in women with gestational DM on medical nutritional therapy or in healthy pregnancies. Pregnant women using insulin had, however, a higher BMI [51]. In two other studies, insulin therapy did not correlate with changes in NT-proBNP concentrations [37, 50].

The effects of clinical variables and pharmacotherapy on BNP and NT-proBNP concentrations are summarized in Table 1.

**Table 1:** The effects of clinical variables and pharmacotherapy on BNP and NT-proBNP concentrations

Clinical parameter	Effect on circulating BNP/NT-proBNP
Age	Increase [23]
Female gender	Increase [23]
African Americans	Decrease [24]
Obesity	Decrease [25–27]
Hypertension	Increase [52]
Coronary heart disease	Increase [53]
Left ventricular systolic dysfunction	Increase [8, 54]
Atrial fibrillation	Increase [55]
Renal insufficiency	Increase [56]
Hepatic disease	Increase [57]
Metformin	Variable [37, 38]
Pioglitazone	Increase [39, 40]
DPP-4i	Unchanged [41]
SGLT2 inhibitors	Decrease [42–47]
GLP-1 RA	Variable [48, 49]
Sulfonylureas	Increase [50]
Insulin	Variable [37, 50, 51]
ACE inhibitors	Decrease [58]
Beta-blockers	Variable [59, 60]

*DPP-4i* dipeptidyl peptidase-4 inhibitors, *SGLT2* sodium–glucose cotransporter 2, *GLP-1 RA* glucagon-like peptide 1 receptor agonist, *ACE* angiotensin-converting enzyme

## NT-proBNP in risk stratification

### Patients without known CVD

The importance of NP in improving the prediction of CV events has been well demonstrated when these biomarkers have been added to multivariate models. A meta-analysis by The *Natriuretic Peptides Studies Collaboration* involving 40 prospective studies from 12 countries and 95,617 general participants, with no history of CVD, demonstrated that NT-proBNP made an additional contribution to conventional risk factors [age, sex, smoking, blood pressure, systolic blood pressure, history of diabetes, and high-density lipoprotein (HDL) cholesterol] in predicting 10-year risk of the composite of CHD and stroke, and the composite of CHD, stroke, and HF [61].

Abnormal NT-proBNP and high-sensitive Troponin T (hs-TnT) levels were able to distinguish individuals with T2DM at high or low CV risk in the ARIC study [14]. An analysis of 42 protein biomarkers in the SUMMIT (*Surrogate markers for micro-and macro-vascular hard endpoints for innovative diabetes tools*) consortium involving individuals with T2DM without apparent CVD and controls, NT-proBNP, high sensitivity troponin T (hs-TnT), and 4 other peptides revealed the ability to increase CV outcome prediction [62].

### Patients with type 2 diabetes and CVD

Among 8401 dysglycemic subjects enrolled in the ORIGIN (*Outcome Reduction with Initial Glargine Intervention*) trial, 59% with prior CVD, NT-proBNP stood out as the main predictor of CV events and death of the 237 evaluated biomarkers [63]. The incorporation of NT-proBNP into the multivariate base prediction model promoted a 39% increase in the net reclassification index for 5-year cardiovascular risk prediction among T2DM patients enrolled in the ADVANCE (*Action in Diabetes and Vascular Disease: Preterax and Diamicron Modified Release Evaluation*) trial [64]. Furthermore, in patients with prediabetes or T2DM and concomitant chronic CHD with normal left ventricular ejection fraction, NT-proBNP levels had a significant association with major adverse CV events (MACE), which was not observed in patients with CHD and normoglycemia [65]. In this study, adding NT-proBNP to the multivariate risk prediction model, which included traditional risk factors, significantly increased the C statistic in patients with prediabetes and T2DM [65].

### Patients with type 2 diabetes and CKD

NT-proBNP above the median was a discriminator of an increased risk for CVD in patients with T2DM and microalbuminuria evaluated in the Steno-2 (*Intensified Multifactorial Intervention in Patients with Type 2 Diabetes and Microalbuminuria*) study [66]. In this study, it was observed that a 10 pg/mL decrease in plasma NT-proBNP during the first 2 years of intervention was associated with a 1% relative reduction in the primary composite outcome of CV death, MI, stroke, heart or leg revascularization procedures, and amputations [66]. The addition of NT-proBNP to a multivariate model improved the prediction of CV outcomes in patients with T2DM and macroalbuminuria enrolled in Sun-MACRO (Sulodexide macroalbuminuria) [67]. In TREAT (*Trial to Reduce Cardiovascular Events with Aranesp Therapy*), which evaluated patients with T2DM, predialysis chronic kidney disease (CKD), and anemia, the addition of NT-proBNP and troponin T to a multivariate model was associated with a net improvement of 17.8% in predicting CV outcomes [68].

The additive predictive value of NT-proBNP has been demonstrated when combined with the coronary artery calcium score (CAC) in patients with T2DM and microalbuminuria [69]. In adjusted continuous analysis, NT-proBNP ≥ 45.2 ng/L and CAC ≥ 400 were equally strong predictors of the composite CV endpoint and death, and there was no interaction between them. While CAC was correlated with underlying ASCVD, NT-proBNP levels were strongly associated with myocardial dysfunction and, probably, other hemodynamic changes [69].

### Mortality in type 2 diabetes

For mortality, some previous studies had also demonstrated the ability of NP in improving the prediction of the multivariable models in patients with T2DM with or without CVD [13, 14, 63].

Tarnow et al. showed that elevated circulating NT-proBNP levels predicted overall and cardiovascular mortality in T2DM patients who were followed at a tertiary clinic [70]. Bruno et al. demonstrated that NT-proBNP was an independent predictor of short-term CV mortality risk in elderly people with type 2 diabetes, including those without preexisting CVD [71]. Recent evidence has revealed the high ability of NP as a single variable in predicting death and CV outcomes. In an analysis of the ELIXA (*Evaluation of Lixisenatide in Acute Coronary Syndrome*) trial, NP alone was as predictive as a multivariable model for death in patients with T2DM recruited within 180 days after an acute coronary syndrome (ACS) [9].

## NT-proBNP in death and CVD prediction

**Fig. 1:**
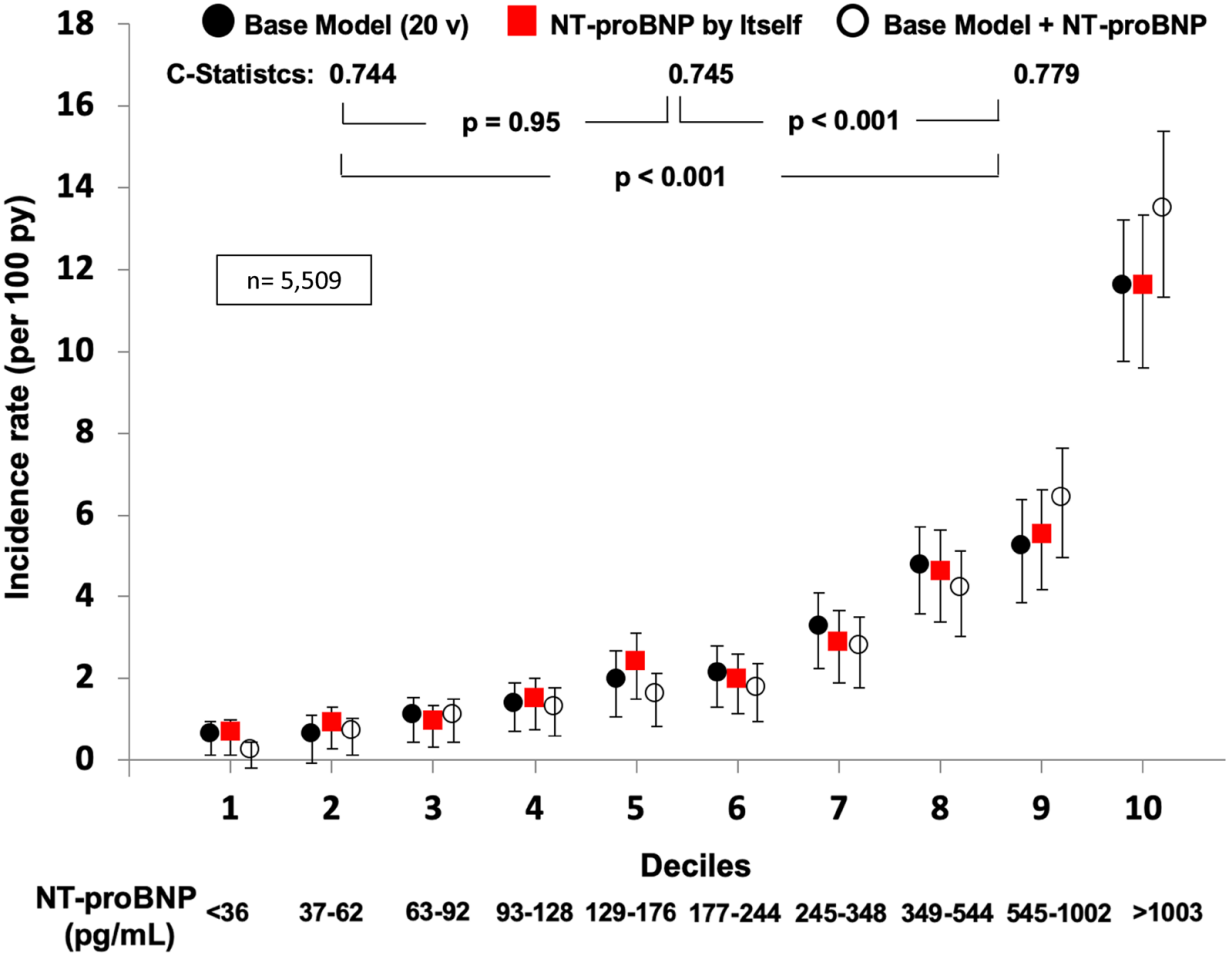
NT-proBNP by itself predicted death as well as the 20 clinical and laboratory variables model. Death prediction models by deciles of predicted risk/deciles of NT-proBNP in high-risk patients with Type 2 Diabetes Mellitus. NT-proBNP: N-terminal pro-B-type natriuretic peptide; py = person/years; Base Model: formed by high sensitivity cardiac troponin, age, albumin, history of heart failure, heart rate, history of stroke, HbA1c, smoking, left ventricular hypertrophy on electrocardiogram (ECG), Q wave on ECG, history of atrial fibrillation, any bundle branch block on ECG, urine albumin-to-creatinine ratio, systolic blood pressure, sex, history of coronary heart disease, low-density lipoprotein cholesterol, estimated glomerular filtration rate, insulin use, and diastolic blood pressure, in decreasing order of X^2^; n = 5509; v = variables. Error bars represent 95% confidence intervals (adapted and reproduced with permission from Malachias et al. [10])

**Fig. 2:**
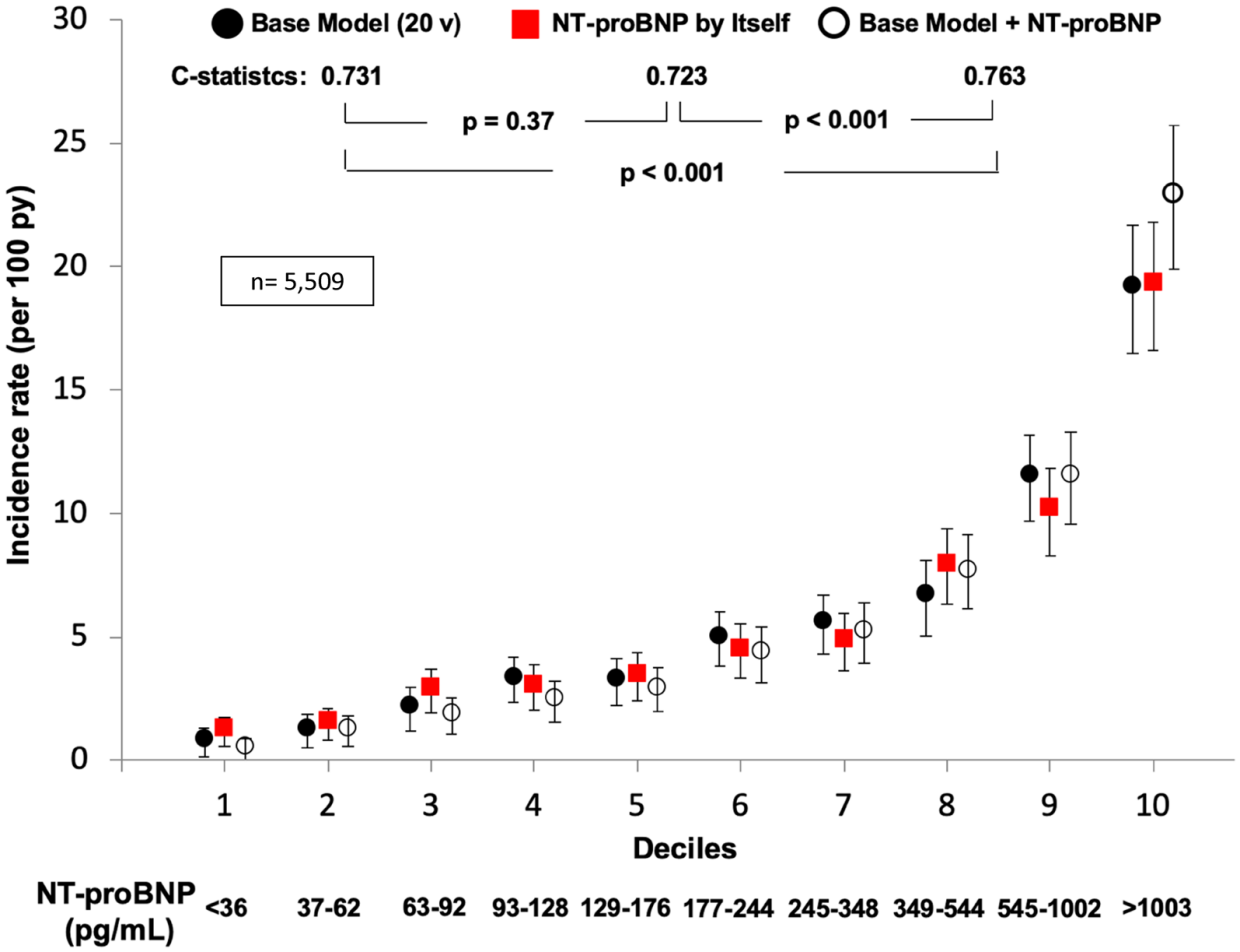
NT-proBNP by itself predicted cardiovascular composite outcome as well as the 20 variables model. Cardiovascular composite outcome (cardiovascular death, resuscitated cardiac arrest, nonfatal myocardial infarction, stroke, or heart failure hospitalization) prediction models by deciles of predicted risk/deciles of NT-proBNP in high-risk patients with Type 2 Diabetes Mellitus. NT-proBNP: N-terminal pro-B-type natriuretic peptide; py = person/years; Base Model: formed by high sensitivity cardiac troponin, history of heart failure, age, albumin, low-density lipoprotein cholesterol, history of atrial fibrillation, history of stroke, systolic blood pressure, HbA1c, smoking, history of coronary heart disease, sex, urine albumin-to-creatinine ratio, any bundle branch block on electrocardiogram (ECG), diastolic blood pressure, insulin use, Q wave on ECG, heart rate, left ventricular hypertrophy on ECG, and estimated glomerular filtration rate, in decreasing order of X^2^; n = 5509; v = variables. Error bars represent 95% confidence intervals (adapted and reproduced with permission from Malachias et al. [10])

We increased knowledge about the discriminatory ability of NT-proBNP as a single variable by analyzing 5509 patients with T2DM and concomitant CVD, CKD, or both, who participated in the ALTITUDE trial [10]. We demonstrated that this cardiac biomarker alone was as predictive as a model composed of the 20 most significant and relevant clinical and laboratory variables in assessing the risk of death from any cause (Fig. 1) and also for the composite CV outcome [CV of death, resuscitated cardiac arrest, myocardial infarction (MI), stroke, or HFH] (Fig. 2) [10]. We also showed that these findings were maintained even in a sensitivity analyses, when patients with a history of HF were excluded or even considering the two main inclusion criteria for the study, CVD or CKD, separately [10]. We further demonstrated that NT-proBNP increases the discriminatory strength for cardiovascular outcomes and death when added to the multivariate prediction model (Figs. 1 and 2) [10].

Subsequent analyses have proven the predictive ability for cardiovascular outcomes and death of NT-proBNP as a single variable among patients with T2DM. Prausmüller et al. [11] analyzed a real-world cohort of 1690 patients with T2DM and demonstrated that NT-proBNP alone had a significantly better predictive ability for both CV death and all-cause death than the proposed risk stratification model by the ESC/EASD [72], as well as the SCORE risk estimate [73]. A recent post hoc analysis of the ORIGIN biomarker study [12], which enrolled patients with dysglycemia, revealed that NT-proBNP as a single biomarker showed discriminatory ability similar to the INTERHEART *(The Effect of Potentially Modifiable Risk Factors Associated with Myocardial Infarction)* risk score [74] to predict CV events or death.

## Are we ready to use NT-proBNP values in death and CV outcomes risk stratification?

Considering the great diversity of available predictive risk scores, their difficulties in use, and limited predictive ability, the idea of using a single biomarker, easy to measure, reproducible, and widely available for risk stratification of patients with T2DM, becomes attractive.

According to the new Universal Definition, HF should be diagnosed by signs and symptoms caused by structural or functional changes, such as dyspnea, and supported by high NP concentrations and/or objective evidence of pulmonary or systemic congestion of cardiogenic origin [8]. About 20–35% of patients with heart failure with preserved ejection fraction (HFpEF) have normal plasma NP concentrations, requiring other tools to establish the diagnosis [75]. However, NP concentrations are usually elevated in patients with HFrEF or even heart failure with mildly reduced ejection fraction (HFmrEF) [8]. Given the high prevalence of underdiagnosed HF in individuals with T2DM, the finding of elevated NT-proBNP may contribute to early diagnosis, as well as the identification of people at higher risk of developing HF due to cardiac stress, in addition to its complications [9–12, 61–71].

Among unselected [11, 14, 74] or high-risk [9, 10] patients with T2DM, elevated NT-proBNP, generally > 125 pg/mL, was associated with an increased risk of CV outcomes and death. This cut-off point was well demonstrated in an unselected cohort of T2DM patients, where NT-proBNP greater than or equal to 125 pg/mL was able to predict unplanned hospitalization for CV events or death in the short term of 12 months [76]. In addition, NT-proBNP < 125 pg/mL had a negative predictive value of 97.6% and a sensitivity of 0.795% to identify individuals who are not at intermediate risk for CV events [76].

In addition, in patients with recent ACS and T2DM, marked increases in BNP or NT-proBNP concentrations were observed in the months prior to HHF. These findings suggest that cardiac deterioration was progressing several months before HF, allowing early identification of the risk of decompensation and the need for hospitalization for HF through periodic monitoring of NP concentrations in high-risk T2DM patients [77].

Considering the negative predictive value of NT-proBNP < 125 pg/mL, this biomarker stands out as a useful tool for initial screening, allowing to distinguish individuals with T2DM at high risk of death and CV events from those at low risk. Life insurers have already identified the value of NT-proBNP measurements in discriminating the risk of death and use this variable to analyze applicants’ proposals to purchase policies [78].

Table 2 presents a summary of the main studies that demonstrated the ability of natriuretic peptides to predict death and CV outcomes in patients with T2DM.

**Table 2:** Natriuretic peptides and prediction of death and cardiovascular outcomes in patients with T2DM

Study or author	Baseline population	Outcome	Follow-up	Cut-off/average	Predictive analysis
Gaede et al. [66]	160 patients with T2DM, age 52–58 y; 60% males; with microalbuminuria	ASCVD	7–8 years	NT-proBNP above and below median 33.5 pg/mL	HR 95% (CI) 3.6 (1.7–7.5)
Tarnows et al. [70]	363 patients with T2DM, age 50–58 y; 72% male caucasians; 6.6% with CHD; 1.5% with HF	CV mortality	9 years	NT-proBNP: T1 < 41 pg/mL vs. T3 > 103 pg/mL	HR 95% (CI) 2.26 (1.27–4.02)
Huelsmann et al. [76]	631 unselected patients with T2DM; age 58.5 ± 13.9 y; 44.7% female; 22.8% with history of any CVD	Unplanned hospitalization for CV events or death	12 months	NT-proBNP > 125 pg/mL	The area under the ROC curve was 0.785 in the prediction of unplanned hospitalizations for CV events or death. The negative predictive value of NT-proBNP < 125 pg/mL for short-term CV events was 98%
Casale-Monferrato et al. [71]	1825 patients with T2DM, age 67.6 ± 10.5 y; no clinical evidence of heart failure	All-cause and CV mortality	5.5 years	NT-proBNP > 91 pg/mL	HR 95% (CI) 2.05 (1.47–2.86) for all-cause death and 4.47 (2.38–8.39) for CV death
ADVANCE [74]	3862 patients with T2DM, 66.9 ± 6.61 y, 61% male	CV events and death	5 years	Log-linear association between NT-proBNP and outcomes	The net reclassification index was increased by 39% with the addition of NT-proBNP to the multivariate risk prediction model for CV events and by 41% for death
SunMACRO [67]	851 patients with T2DM and nephropathy, 64 ± 9 y, 76% males	Renal and CV events	Mean (SD) follow-up was 11.2 (6.6) months in the sulodexide group and 10.7 (6.6) months in the placebo group	NTproBNP > 407 pg/mL for CV outcome, > 973 pg/mL for renal outcome	C statistic for CV events was improved by adding NT-proBNP to the multivariable model (0.722 vs. 0.658, P = 0.018)
ELIXA [9]	5525 patients with T2DM and acute coronary event-related hospital admission within 180 days. Placebo group 60.6 ± 9.6 y, intervention group 59.9 ± 9.7 y, 69.3% males, 75.2% whites	CV death, MI, stroke, or hospitalization for unstable angina	26 months	Group without CV events: BNP = 95 (92–98), NT-proBNP = 285 (274–295) vs. group with CV events: BNP = 198 (184–213), NT-proBNP = 703 (644–766) (pg/mL)	BNP or NT-proBNP alone predicted death equally well as all other variables combined (C-statistics: 0.77 vs. 0.77)
ALTITUDE [10]	5509 high-risk patients with T2DM, 64 ± 6.8 y, 67% males 56% whites	All-cause death, CV composite outcome	2.6 years	NT-proBNP deciles	NT-proBNP by itself was similar to a 20-variable model in predicting both death and CV events
Prausmüller et al. [11]	1690 patients with T2DM, 63 y, 54% male, 10 y of T2DM duration	CV and all-cause death and CVD and all-cause hospitalizations	10-year follow up for fatal CVD and all-cause death and a 5-year follow up for CVD and all-cause hospitalizations	NT-proBNP > 125 pg/mL, and NT-proBNP tertiles (1st tertile: 59 pg/mL [IQR 59–59], 2nd tertile: 122 pg/mL [IQR 90–156], 3rd tertile: 376 pg/mL [IQR 267–648])	NT-proBNP was superior to the ESC/EASD risk model for all outcomes (C-index: CVD hospitalization: 0.74 vs. 0.54; all-cause hospitalization: 0.62 vs. 0.55; p < 0.001 for all comparisons)
ORIGIN [12]	8401 people with CV risk factors plus impaired fasting glucose, impaired glucose tolerance, or T2DM, 63.2 ± 7.9 y, 66.1% males	CV composite outcome (myocardial infarction, stroke, HFH, and CV death), all-cause death, and CV death	6.2 years	NT-proBNP categories (< 128; 128–401; 402–808, 809–1730, > 1740 pg/mL)	For each increase in NT-proBNP by one level the HR increased 53% for the composite CV outcome, 48% for death, and 65% for CV death. The C-statistic of NT-proBNP by itself was similar to that of the multivariate model for any outcome

*T2DM* type 2 diabetes mellitus, *ASCVD* combined endpoint for cardiovascular disease comprising cardiovascular mortality, nonfatal myocardial infarction, nonfatal stroke, percutaneous coronary interventions, coronary artery bypass graft, vascular surgery, and amputations, *HHF* heart failure hospitalization, *y* years old, *CV* cardiovascular, *CVD* cardiovascular disease, *HR* hazard ratio, *IQR* interquartile range, *NT-proBNP* N-terminal pro-B-type natriuretic peptide, *BNP* B-type natriuretic peptide

## Limitations

Although the benefit of using NT-proBNP and BNP in the context of HF has been demonstrated and, more recently, expanded to include risk stratification of patients with T2DM, some limitations to its use in clinical practice should be highlighted. NP may rise in the presence of anemia, CKD, advanced age, left ventricular hypertrophy, myocardial ischemia, atrial fibrillation, stroke, increased heart rate, volume overload, and drugs such as beta-blockers [18], while lower concentrations may occur in obese [32, 34] and African American individuals [31]. Therefore, interpretation of test results needs to take into consideration several clinical characteristics as well as ongoing pharmacotherapy. Most importantly, more data from dedicated trials are needed to ascertain whether patients with T2DM and elevated NP levels benefit from specific therapies to a larger extent than patients with T2DM who have normal NP levels.

## Conclusions

Recent evidence of the discriminatory strength of NT-proBNP as an independent predictor of death and CV outcomes calls attention to the possibility of broader use of this simple, easy to measure, and widely available resource to improve risk stratification, for patients with T2DM. Although more evidence is needed and this tool is not yet recommended by guidelines, the incorporation of NT-proBNP measurement should be strongly considered when evaluating patients with T2DM in clinical practice. Ongoing studies should better define the role of this biomarker in the assessment, follow-up, and treatment of patients with T2DM.

## Abbreviations

ACC: American College of Cardiology
ACE: Angiotensin-converting enzyme
ACS: Acute coronary syndrome
ADA: American Diabetes Association
ADVANCE: Action in Diabetes and Vascular Disease: Preterax and Diamicron Modified Release Evaluation
AHA: American Heart Association
ALTITUDE: The Aliskiren Trial in Type 2 Diabetes Using Cardiorenal Endpoints
ANP: Atrial natriuretic peptide
ARIC: The Atherosclerosis Risk in Communities
ASCVD: Atherosclerotic cardiovascular disease
BMI: Body mass index
BNP: B-type natriuretic peptide
CAC: Coronary artery calcium score
CANVAS: Canagliflozin Cardiovascular Assessment Study
CHD: Coronary heart disease
CKD: Chronic kidney disease
CV: Cardiovascular
CVD: Cardiovascular disease
DAPA-HF: Dapagliflozin and Prevention of Adverse Outcomes in Heart Failure
DM: Diabetes Mellitus
DNP: Dendroaspis natriuretic peptide
EASD: European Association for the Study of Diabetes
ECG: Electrocardiogram
ELIXA: Evaluation of Lixisenatide in Acute Coronary Syndrome
EMPEROR-Reduced: Empagliflozin Outcome Trial in Patients with Chronic Heart Failure with Reduced Ejection Fraction
EPIC: The European Prospective Investigation into Cancer and Nutrition
ESC: The European Society of Cardiology
FRS: Framingham risk score
GLP-1 RA: Glucagon-like peptide 1 receptor agonist
HbA1c: Glycated haemoglobin
HFpEF: Heart failure with preserved ejection fraction
HFmrEF: Heart failure with midrange ejection fraction
HFpEF: Heart failure with preserved ejection fraction
HHF: Heart failure hospitalization
HR: Hazard ratio
hs-TnT: High sensitivity troponin T
INTERHEART: The Effect of Potentially Modifiable Risk Factors Associated with Myocardial Infarction
IQR: Interquartile range
MACE: Major adverse cardiovascular events
MI: Myocardial infarction
NP: Natriuretic peptides
NPR: Natriuretic peptide receptor
NPRA: Natriuretic peptide receptor coupled to guanylyl cyclase A
NPRB: Natriuretic peptide receptor coupled to guanylyl cyclase B
NPRC: Natriuretic peptide clearance receptor
NT-proBNP: N-terminal pro-B-type natriuretic peptide
ORIGIN: Outcome Reduction with Initial Glargine Intervention
POCT: Point-of-care testing
PONTIAC: NT-proBNP selected prevention of cardiac events in a population of patients with diabetes mellitus without a history of cardiac disease
py: Person/years
RAAS: Renin–angiotensin–aldosterone system
ROC: Receiver operating characteristic
SAT: Subcutaneous adipose tissue
SAVOR: Saxagliptin Assessment of Vascular Outcomes Recorded in Patients with Diabetes Mellitus
SCORE: The Systematic Coronary Risk Evaluation
SGLT2: Sodium–glucose cotransporter 2
Steno-2: Intensified Multifactorial Intervention in Patients with Type 2 Diabetes and Microalbuminuria
SUMMIT: Surrogate markers for micro-and macro-vascular hard endpoints for Innovative diabetes Tools
T2DM: Type 2 diabetes mellitus
TREAT: Trial to Reduce Cardiovascular Events with Aranesp Therapy
UKPDS: The United Kingdom Prospective Diabetes Study
VAT: Visceral adipose tissue areas
Y: Years old
